# A model-based approach for simulating adaptive clinical studies with surrogate endpoints used for interim decision-making

**DOI:** 10.1016/j.conctc.2020.100562

**Published:** 2020-04-19

**Authors:** Xiaotian Chen, Alan Hartford, Jun Zhao

**Affiliations:** a Data and Statistical Sciences, AbbVie Inc, North Chicago, IL, United States; b Statistical and Quantitative Sciences, Takeda Pharmaceuticals Inc, Cambridge, MA, United States; c Data Science, Astellas Pharma Global Development, Northbrook, IL, United States

**Keywords:** Adaptive design, Bayesian model, Combination test, Surrogate marker, Survival analysis, Historical data

## Abstract

In clinical trials, when exploring multiple dose groups to establish efficacy and safety on one or more selected doses, adaptive designs with interim dose selection are often used for dropping less effective dose groups. When it takes a long time to observe primary outcomes, utilizing information on a surrogate endpoint available at an earlier interim may be preferred for selecting which dose to continue. We propose a Bayesian model-based approach where historical data can be leveraged to incorporate a correlation model for investigating the design's operating characteristics. Simulation studies were conducted and the method can be readily applied for power and sample size calculations.

## 1 Introduction

In drug development, it is critical to get the dose right. Clinical programs fail when either the dose is too low to achieve efficacy or too high resulting in toxicity or adverse reactions. When moving a drug development program into phase 3, the risk of choosing a wrong dose may be mitigated by bringing more than one dose forward. However, studying more than one dose in large confirmatory studies requires a larger sample size, takes longer, and is more costly. Adaptive designs can be used to select doses at an interim analysis so that less efficacious or toxic doses can be dropped while moving forward with more appropriate doses if they exist. Such a design usually has advantages in not only statistical, but also in an ethical perspective.

When it takes a long time to observe the primary endpoint, e.g., a time-to-event (TTE) endpoint with long follow-up, utilizing information on an early surrogate endpoint at an interim analysis (IA) can be more efficient for selecting a dose to continue. For instance, observing confirmed/sustained disability progression in subjects with multiple sclerosis, based on a composite primary endpoint EDSS+ (Expanded Disability Status Score Plus) [1,2] may take several years. In such a situation, it is appealing to use outcomes with relatively short follow-up that are closely related to the primary endpoint for early decision-making, e.g., dose selection.

Among the literature of surrogacy in the past several decades, Prentice [3] provided a formal description of perfect surrogacy. It has been extended to measure the proportion of the treatment effect explained by the surrogate variable [4,5]. Other causal frameworks are also considered [[6], [7], [8], [9], [10], [11]]. From a design perspective, incorporating adaptation with decision rules, there are many examples in a wide range of scenarios where analyses can be conducted at an IA utilizing early or intermediate assessments [[12], [13], [14], [15], [16], [17], [18], [19], [20]]. When a surrogate endpoint is used to make an interim decision, risk may arise about how well the surrogate endpoint predicts the primary outcome. Therefore, the correlation between the primary endpoint and the surrogate endpoint should be understood and incorporated properly to evaluate dose groups at an IA. Friede et al. [12] considered simulating correlated standardized test statistics from a multivariate normal distribution. Carreras et al. [19] created assumptions based on copula functions to relate the primary endpoint and the surrogate variable. However, choice of measures such as correlation and concordance, is often subjective in current practice.

Current software packages, e.g., ADDPlan (ICON plc. ADDPLAN Software), requires input of both the correlation and the concordance. Here, the concordance is the proportion of simulated studies where the results of the two endpoints agree on whether the simulated study is positive or negative in regard to a common α. When study designs are evaluated through the inputting of assumed correlation or concordance, it is found that specifying the latter measure between the surrogate marker and the primary endpoint has a dominant impact resulting in the correlation, which could be well studied from prior historical data and contains important information, being suppressed. This can be easily shown via ADDPlan by running simulations varying both correlation and concordance. Additional challenges also exist when the data of interest contain incomplete observations, e.g., censored TTE data, where it's difficult to simulate correlated data based on a given correlation or concordance value. Therefore, in some circumstances, specifying a single value measure of correlation or concordance between the two endpoints may not be considered sufficient. In this paper, we propose a model-based approach for simulating adaptive clinical studies with surrogate endpoints used for interim decision-making. Using this model-based approach, specifying the concordance is not needed and the correlation is specified within the model structure assumed. However, care must be taken when handling the sensitivity of the relationship between the surrogate endpoint and the primary endpoint. To this end, Bayesian models can be adopted to account for the variability in the model parameters to better facilitate the treatment selection decision.

When designing a trial whose primary outcome is a TTE endpoint with a long follow-up period, or is any type of delayed response, its linkage with the short-term surrogate is worth investigating. Even though the possibility is sometimes allowed to evaluate the primary outcome and the short-term surrogate jointly in an on-going basis during the trial, the direct information on the long-term primary endpoint may not be accumulated enough for sufficient inference. At the design stage, with the assistance of historical clinical trial data, either the data from the earlier development program or shared placebo data from the therapeutic research area, the relationship between the primary endpoint and the early surrogate can be studied from reasonably appropriate sources of information. In the past few decades, the idea of leveraging such historical data in control arms (placebo or standard of care) in similar populations has proved to be useful when the borrowed information is handled properly [21,22]. We will leverage historical data to model the relationship between the endpoints, which will play a key role in setting up the IA decision rule that usually needs to be specified prior to the conduct of the study.

For simplicity to illustrate designing a clinical trial with a planned IA to choose arms to move forward in the study, we will consider the trial to have two stages. In stage 1, subjects are randomized to all treatment groups. By the time of a planned interim analysis, those who have complete information on the early surrogate endpoint will contribute to the decision-making for selecting the targeted investigational dose(s) that warrant further development to move forward to stage 2. Incorporating the seamless phase II/III design framework, two stages of development can be combined, i.e., a learning phase and a confirmatory phase [23,24]. At the end of the trial, a final analysis can be performed based on the primary endpoint, combining the information from both stages. We consider the final analysis to be a frequentist analysis, such as hypothesis testing, which is a common practice in a confirmatory trial setting.

In this paper, we first describe the method of the proposed Bayesian model-based approach in Section 2. In Section 3, we advance to apply the method to a trial design and simulate scenarios of interest. We provide discussion in the final section.

## 2 Model-based method: a bayesian approach

### 2.1 Modeling correlation using historical data

Instead of using a single value to describe the correlation between the primary endpoint and the early surrogate endpoint, we can fit a model incorporating historical data. There has been a variety of modeling approaches proposed in the literature for borrowing from a single historical study or multiple historical studies. Without loss of generality, in this paper we use a single historical dataset, including the information for both the primary outcome and the early surrogate endpoint. The analysis population of the historical dataset should, and so is assumed to be in this paper, a match with the targeted population of interest for clinical study to be designed.

Considering a continuous surrogate endpoint *X*, when the primary endpoint *T* is a TTE outcome that takes a long time to observe, an appropriate model, for example, a log-normal model, can be fitted on the historical dataset.

(1) log(T)=bX+a+ε,

where $\varepsilon \sim N\left(0,\sigma^{2}\right)$.

Based on the assumed log-normal model, a non-informative prior distribution or a properly justified informative prior distribution can be imposed on (a,b,σ). Then the model is fitted to the historical data and we can obtain MCMC samples from the posterior distribution of (a,b,σ). The choice of prior for σ controls the variability of the correlation. The rest of the design procedure will be demonstrated in detail in Section 3. One of the benefits of Bayesian modeling is the ability to take full account of the uncertainties related to the model parameters and thus the uncertainty in the correlation between the surrogate and the primary endpoint in this case. The error term ε in the considered log-linear model is a very important factor and it plays a key role in galvanizing the variability of the correlation and the joint distribution of the two endpoints. It is well known that in the frequentist analogue regarding the log-linear model, the distribution of the primary endpoint *T* depends on the distribution of the error term. When denoting ε=σW, other than the log-normal model that we have considered, a standard extreme value distribution of *W* will give *T* a Weibull distribution while a standardized logistic distribution of *W* will give *T* a general log-logistic distribution. Our proposed Bayesian model inherits the same regression model structure and further allows specification of prior distributions on the model parameters. When noninformative priors are used for the model parameters, the inference based on the posterior distributions will be consistent with those based on the maximum likelihood estimates of the parameters in the frequentist context. In situations where there is prior belief on the parameters, informative priors can also be specified to incorporate the prior information.

We can follow a similar framework when the primary endpoint is a continuous outcome or a binary outcome. A standard Bayesian linear model or a logistic model may be utilized, respectively.

### 2.2 Specifying the interim decision rule

The interim analysis and its timing need to be preplanned. When the predefined number of subjects achieve maturation of the surrogate endpoint, an interim analysis will be performed to select dose(s) and move to the next stage. In most cases, there is no timing gap or stopping on recruitment when an interim analysis is performed. In the interim analysis, all the data before the cut-off date should be included in the statistical analysis. However, we expect the number of mature observations on the primary endpoint will be low at the IA. Therefore, the decision-making for treatment selection must rely solely on the surrogate endpoint. Through prestudy simulation and its operating characteristics based on the model fitting that we introduced in the previous section, the posterior distribution of the model parameters can prospectively help us develop a decision rule at the IA to select dose groups based on stage-1 surrogate endpoint observations.

Based on the model fitting and the existing understanding of the clinical background, if a higher value in the surrogate endpoint indicates improvement in the primary endpoint, one can select the observed best dose at the IA to carry forward to the next stage if the observed difference in group means of the surrogate endpoint exceeds a certain threshold [25]. Alternatively, some variation or extra criteria can also be used, e.g., the best arm needs to be at least 50% better than the second best for it to be moved forward. In addition, it could be more than one arm that is allowed to be selected to move forward to the next stage, or none, the latter effectively serving as a futility criterion.

In the prestudy model fitting utilizing the historical data, it is recommended that the posterior distributions of the model parameters be examined to ensure valid statistical inference regarding the use of a surrogate for evaluating treatment effect through its relationship with the unobserved primary endpoint at the IA, e.g., whether the 95% credible interval of the slope in the log-linear model covers zero. Note that the interim decision rule and all the relevant design parameters are needed to be clearly specified prior to the start of the study.

### 2.3 Multiplicity adjustment for testing

When there are multiple treatment arms and hence more than one hypothesis to test in a clinical trial, especially in a confirmatory trial, the familywise error rate (FWER) needs to be controlled. In the proposed adaptive design setting where there are multiple dose groups in the study, an unblinded IA is conducted at the end of stage 1 and the final analysis will be performed based on the primary endpoint. The FWER can be controlled when a combination test with closed testing procedure is used at the time of final analysis.

Suppose in the study there are initially k active arms and one control arm. Let $S_{1}=1,\ldots ,k$ denote the index set of elementary null hypotheses. It is well recognized that the closure principle [26,27] to construct multiple test procedures can control the FWER in the strong sense. Based on $H_{i}$, where $i\in S_{1}$, the closed test procedure first constructs all intersection hypotheses $H_{S}$,

$$H_{S}=\underset{i\in S}{\cap }H_{i},S\subseteq S_{1},$$

For each intersection test, the multiplicity within each stage is addressed by using any standard multiple test procedure and the adjusted p-values can be calculated accordingly. Then the stagewise p-values from stage 1 and stage 2 will be combined [12,25,28] through a prespecified combination function. A two-stage combination function $C\left(p_{1},p_{2}\right)$ is monotonically increasing in both arguments and the null hypothesis will be rejected if $C\left(p_{1},p_{2}\right)<c$, where *c* is the critical value for that corresponding combination function at a certain significance level of α. A well-known and widely used combination function is the weighted inverse normal combination function [29,30].

$$C\left(p_{1},p_{2}\right)=1-\text{Φ}\left[w_{1}{\text{Φ}}^{-1}\left(1-p_{1}\right)+w_{2}{\text{Φ}}^{-1}\left(1-p_{2}\right)\right],$$

where $w_{1}$ and $w_{2}$ denote the prespecified weights such that $w_{1}^{2}+w_{2}^{2}=1$. Φ is the cumulative distribution of the standard normal distribution. When the information fraction based on the preplanned sample size is considered, the weights are $w_{1}=\sqrt{n_{1}/\left(n_{1}+n_{2}\right)}$ and $w_{2}=\sqrt{n_{2}/\left(n_{1}+n_{2}\right)}$.

To reject an individual elementary null hypothesis $H_{i},i\in S_{1}$, all the relevant intersection hypothesis need to be rejected by their α level combination tests. For example, when there are three elementary null hypotheses $H_{1},H_{2},H_{3}$, the intersection hypotheses regarding $H_{1}$ are $\left\{H_{1},H_{1}\cap H_{2},H_{1}\cap H_{3},H_{1}\cap H_{2}\cap H_{3}\right\}$ and each one in this set needs to be rejected in order for $H_{1}$ to be rejected. For the intersection test that involves active arms which are dropped at IA, the corresponding stage-2 p-value that will be plugged into the combination function only takes into account the active arms which are moved forward [25,31]. That is, the stage-2 p-value is set to be

$$p_{2,S}=p_{2,S\cap S_{2}},$$

where $S_{2}$ is the index set of the selected active arms at interim. $p_{2,\emptyset }$ is set to 1.

The combination of stagewise p-values for each intersection hypothesis, for most multiple testing procedures within stage, are built upon the standardized Z statistics from each of the k comparisons in each stage. The calculation of such Z statistics is straightforward for continuous and binary endpoints. As for TTE outcome, the logrank test can be performed and is considered in this paper. Regarding each comparison (active arm vs control), let $U_{1}$ be the unstandardized logrank statistic based on the data of primary endpoint at interim and let $V_{1}$ denote the variance of $U_{1}$. Similarly, let $U_{2}$ denote the unstandardized logrank statistic based on the full data of primary endpoint from all subjects and $V_{2}$ be the variance of $U_{2}$. The stage-1 standardized statistic is calculated by $Z_{1}=U_{1}/\sqrt{V_{1}}$. Based on the independent increment property, the stage-2 standardized statistic can be calculated by $Z_{2}=\left(U_{2}-U_{1}\right)/\sqrt{V_{2}-V_{1}}$. Then the rest of the calculation for the p-values and the combination test will follow the procedure introduced earlier in this section.

Note that the interim analysis is used to make a decision about which arm or arms to move forward with based on the biomarker but the p-values combined at the end of the study should only be for the primary endpoint [25]. Even though we don't have much mature data for the TTE endpoint at the interim, the p-value for only these data is the stage-1 p-value while the majority of the information on the primary endpoint are included in stage-2 p-value.

## 3 Application in designing a phase 2/3 study

In this section, we will apply the method described in the previous section to design a phase 2/3 study utilizing a surrogate endpoint to select dose(s) at the end of stage 1 with the primary clinical endpoint being the time to event endpoint.

### 3.1 Endpoints and population

In neurology, multiple sclerosis is a chronic autoimmune and neurodegenerative disorder of the central nervous system (CNS) that is characterized by inflammation, demyelination, axonal transection, and neuronal loss. In clinical development, a composite endpoint EDSS+ is proposed to be utilized to detect disability improvement (functional improvement as opposed to disability progression) in subjects with multiple sclerosis. We consider a circumstance where the primary endpoint of the study is time to first 6-month-confirmed disability improvement on any of the EDSS + components: EDSS (Expanded Disability Status Score); T25FW (25-foot timed walk); and 9HPT (9-hole peg test). The shortfall of applying such a composite time-to-event endpoint is that the treatment duration might be very long (i.e., 2–3 years) in order to collect enough events with a long confirmation period. Therefore, we may consider a surrogate endpoint, which can be observed at an earlier time point. In recent clinical development, researchers are considering an endpoint which is a function of the components at each visit, called Overall Response Score (ORS). This endpoint may be sensitive to detect a treatment signal in less time, e.g., ORS at Week 24 (6 months) or Week 48 (12 months). Intuitively, this endpoint is considered to be highly correlated to the primary endpoint EDSS+, since both endpoints are derived from the longitudinally collected domain scores. The ORS is the sum of the indicators of improvement (+1) or worsening (−1) at 4 domains (EDSS, T25FW, 9HPT-Right hand, 9HPT-Left hand), and may be observed in a relatively short time without the need of a confirmation period. A higher ORS score is perceived to be associated with a shorter time to confirmed improvement, while a lower ORS score is perceived to be associated with a shorter time to confirmed worsening. We will illustrate how the proposed approach can be utilized to design a study and facilitate the interim decision-making.

Since the endpoints are composite scores from multiple components or domains, the patient population should be the ones that have at least one domain at risk. Normally, the patient population includes at least one domain deficiency in order to detect disability improvement. It is also likely to be more sensitive to detect disability progression for the patient population with at least one domain deficiency. Therefore, when historical data are included as the prior information, the patient population should be very similar to the targeted phase II/III patient population.

### 3.2 Finding the correlation between endpoints

For subjects with multiple sclerosis, normally the treatment duration will be 96–156 weeks (2–3 years) for the primary TTE endpoint T. The events need to be confirmed following an additional 6-month confirmation period. The surrogate endpoint X (treated as continuous with scale [-4, 4]) is believed to be correlated with the primary endpoint and it may take less than one year to observe.

We can use a historical dataset to study the relationship between the primary endpoint (time to confirmed improvement based on EDSS+) and the early outcome (ORS). The data can be from the placebo arm of a historical study or an open database, e.g., from the MS consortium MSOAC. A subset of the data is used in order to mimic the patient population of interest. Here for illustration only, we are using a simulated dataset in this section to mimic this real historical dataset. The Bayesian log-normal model can then be fitted where non-informative priors are used for the model parameters. The log-normal scale parameter σ is assumed to follow noninformative Gamma(0.001, 0.001) distribution, which is widely used as a noninformative prior in Bayesian survival analysis. For the prior of the regression coefficients, i.e. the intercept and the slope, noninformative uniform improper distribution is assumed. In the absence of further clinical knowledge, a homogeneous relationship between the early surrogate outcome and the primary outcome is assumed across all active arms. Plugging in the posterior mean of the parameters, the fitted model is

(2) log(T)=−1.37X+0.16

The posterior distribution of the standard deviation of the noise term can also be obtained and the mean is 1.53. Also note that the 95% credible interval of the slope parameter is [−1.53,−1.22], which excludes zero. This supports the validity of the use of this model.

### 3.3 Study design elements

**Fig. 1:**
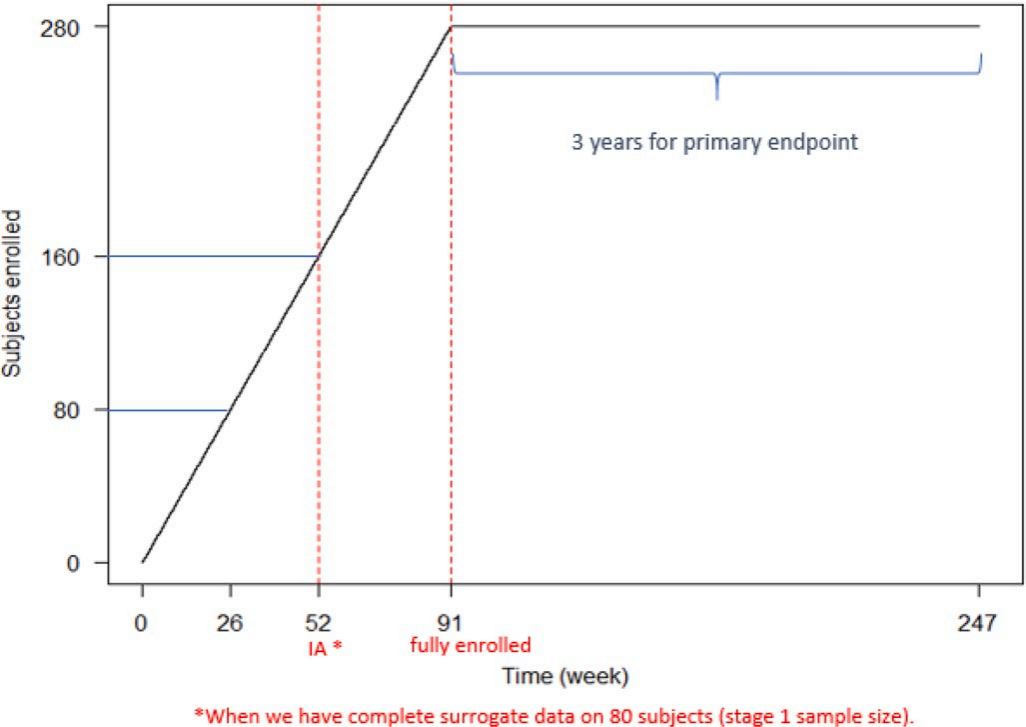
Expected Subjects Recruitment (e.g. targeting total of 280 subjects to enroll).

Assume that we are designing a trial where three active arms (low dose, medium dose and high dose) are investigated versus a control arm. One IA is planned at the time of 20 subjects/arm (80 in total) exposed for 26 weeks when their surrogate observations are complete. For illustration, under a simplistic constant enrollment speed assumption, the projected timing of the IA is around week 52. Fig. 1 shows the recruitment with a constant enrollment speed. After the IA, the newly enrolled subjects will only be assigned to the selected active arm(s) as well as the control arm.

The objective of the IA is to select an arm to be carried forward to the next stage. The decision rule will be based on the observed surrogate values. Let $X_{ij}$ denote the value of surrogate variable for subject *j* in arm *i*, where i=0,1,2,3 with regards to the placebo, low dose, medium dose and high dose arms, respectively; $j=1,\ldots ,n_{i}$. The $n_{i}$ denotes the number of subjects with complete surrogate observation at IA in arm *i*. In this considered example, $n_{i}=20$ for each arm. Let ${\bar{X}}_{i}$ be the group mean for arm *i* and ${\bar{X}}^{max}={\text{max}}_{i\in \left\{1,2,3\right\}}{\bar{X}}_{i}$ is the largest observed group mean among the active arms. The IA will result in either one of the following situations:

• Select the best treatment arm (in terms of group means) to bring into stage 2 (along with the control arm) if the corresponding observed difference in group means of the surrogate endpoint exceeds a threshold Δ, i.e. select the best treatment arm as well as the control arm if ${\bar{X}}^{max}-{\bar{X}}_{0}\geq \text{Δ}$.
• If none of the treatment arms cross the threshold in the surrogate endpoint, stop the trial for futility.

Simulations will determine the threshold, as a design parameter, at the study design stage. We will illustrate how this threshold value can be selected from a candidate set of clinical interest.

Assuming the log-rank test will be used in the final analysis where the inverse normal combination tests are performed. The power will be defined as rejecting at least one hypothesis regarding the comparison of active arm versus control. The within-stage multiplicity is addressed by adopting Dunnett's test.

In literature, subjects that belong to the dropped arms may be withdrawn from the current treatment and given other therapies. This is a situation where their primary endpoint data will not be included in the final analysis [12]. Alternatively, these subjects can remain on the original treatment through the rest of the study and contribute to the final analysis on the primary endpoint, as is considered in the study example in this paper. It is important to note that one should determine whether or not such subjects should continue in their originally assigned treatment arms and should prespecify this in the study protocol. Although the resulting data are not needed for the final analysis for the primary endpoint, there may be other reasons for continuing to treat and follow them, i.e., for accumulating additional safety data or for ethical reasons.

Although the early surrogate ORS takes a much shorter time to be observed than the primary endpoint, it is still a response in a delayed fashion in this multiple sclerosis study example. Note that pausing the enrollment is most often not practical, e.g., as it would risk engagement with the sites for completing the study. Therefore, a small portion of subjects will still be randomized to the arms that will be dropped at the IA before their surrogate endpoint matures by the time of the IA. Although these subjects could still be followed through the rest of the study, they will not contribute to the final analysis at the end of the study. When the enrollment is slow, there will be very few such subjects. We will further discuss this later.

### 3.4 Simulation for study design

In simulation, for each arm, the event times are first generated marginally based on the assumed event rates of interest at the end of 3 years that are projected by the study team. The widely used exponential distribution is considered in this case. Then based on the fitted model that we described earlier, the surrogate marker can be simulated using $X=\left(\text{log}\left(T\right)-a\right)/b+\varepsilon^{'}$ where $\varepsilon^{'}=-\varepsilon /b$ and ε is from equation (1). In this way, there is no direct assumption of the treatment difference on the surrogate endpoint. As compared to software packages, e.g., ADDPlan, that require assumption of a concordance rate between the primary endpoint and the surrogate marker of how often they both result in a positive trial, this model-based approach only requires a correlation be assumed indirectly by specifying the treatment difference for the primary endpoint and the variance of the random error, ε. The treatment effect of the surrogate results from the model. By the nature of the simulation process, the simulation order of the primary endpoint and the surrogate does not need to be the same as how they are observed during the course of the trial. In the simulation process, we focus on the association between the endpoints derived from the earlier fitted model, instead of treating one endpoint as an independent variable and the other as the dependent variable. Due to a lack of background knowledge of the surrogate variable upfront in terms of its distribution and treatment effects across dose groups, the primary endpoint is first generated based on the target product profile (TPP) and relevant assumptions that are desired to be investigated and the surrogate variable is subsequently simulated based on the earlier fitted model. To take into account the variability in the association between the primary endpoint and the surrogate, the above procedure is replicated 5000 times (simulating 5000 trials) using the posterior draws of the model parameters.

**Fig. 2:**
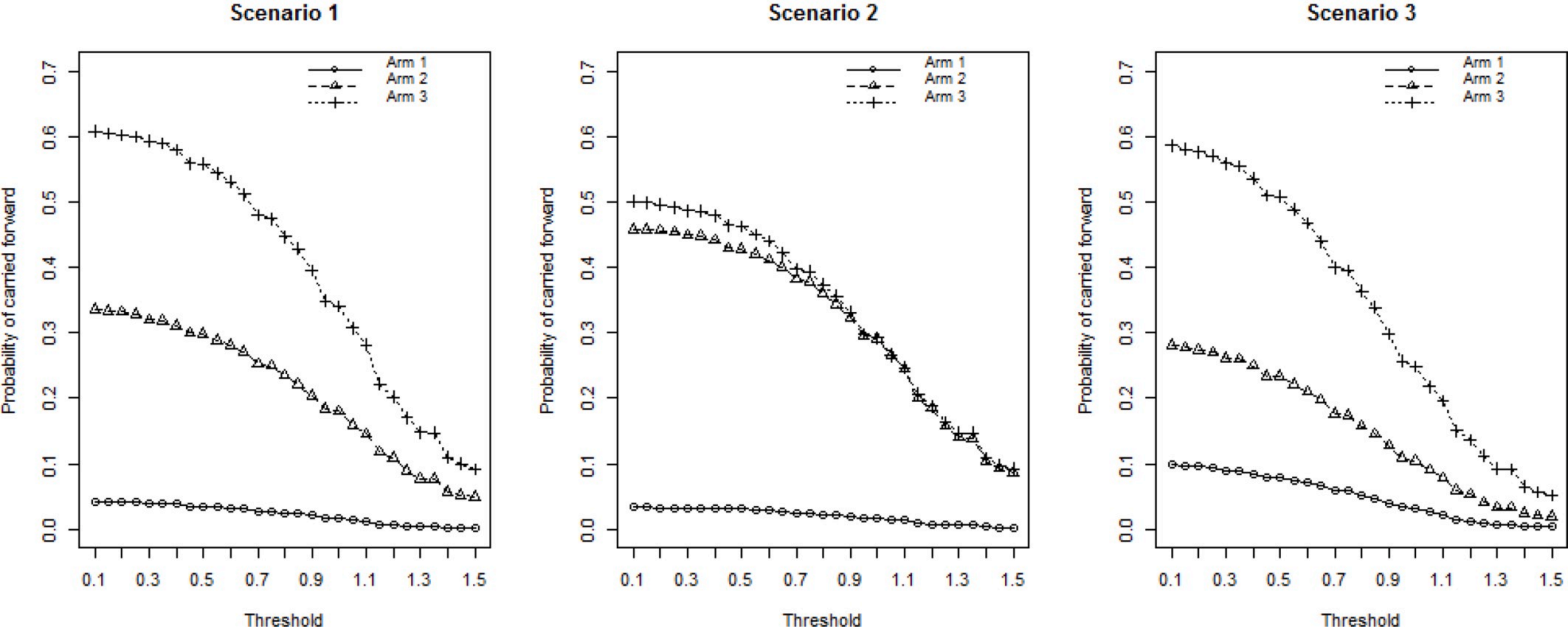
Probabilities for each arm to be carried forward to stage 2.

**Fig. 3:**
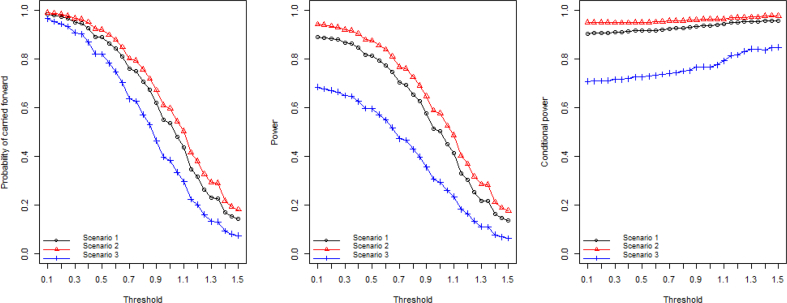
Probability of the study being carried forward to stage 2; Overall power of the study; Conditional power given the study is carried forward to stage 2.

We start with simulating three scenarios of event rates of interest (by the time of 3 years) for the primary endpoint. We assume for Control arm, Low dose, Medium dose, High dose:

• Scenario 1: 10%, 15%, 25%, 30%;
• Scenario 2: 10%, 15%, 30%, 30%;
• Scenario 3: 10%, 15%, 20%, 25%;

A sample size of 20 subjects per arm in stage 1 and 100 subjects per arm in stage 2 is considered. An overall 5% dropout (by 3 years) is assumed. The operating characteristics of the simulation results for the above three scenarios are presented in Fig. 2 and Fig. 3. Threshold values on the treatment difference from 0.1 to 1.5 on the surrogate endpoint at IA are considered based on clinical interest. It is clear that the threshold has an impact on the operating characteristics. The experimental arms have less chance to be moved forward as the threshold increases. The arm with the largest effect size in scenario 1 is carried forward around 60% of the time when using a small threshold, and it drops to about 10% when a stringent threshold is used. The high dose in scenario 3 also has around 60% probability under a small threshold to be selected even though its treatment effect is smaller than that of the high dose in scenario 1. This is primarily due to the presence of a less competitive medium dose in scenario 3 (event rate of 20%), compared with its counterpart in scenario 1 (event rate of 25%). With stringent thresholds, experimental arms with larger effect size are still chosen more often than those with smaller effect size.

The high dose and medium dose in scenario 2 share the same true treatment effect and both have around 50% chance to be carried forward. They have equal chance to be moved forward when only one of them will be allowed to be continued according to the prespecified rule at the IA. The low dose with 15% event rate is selected less than 10% of the time, which is a desired consequence because the treatment effect is highly unlikely to be clinically meaningful. For scenario 2, the study is carried forward to stage 2 more often than the other scenarios. This is because of the underlying high treatment effect in the medium dose and the high dose. The study has 99.2% probability of moving forward when a minimal threshold of 0.1 is used. The overall statistical power shows a similar pattern where scenario 2 gives the best power ranging from 90% to 20% as the threshold increases.

The power in scenario 3, on the contrary, is relatively low due to the small effect size and the resulting high probability of futility stopping at IA. The conditional power, given the study is carried to stage 2, benefits from the setting with more stringent thresholds at the IA. The moderate increase in the conditional power adequately reflects the advantage of the use of threshold as a “screening” step for evaluating experimental arms where more promising arms are selected into the next stage.

**Fig. 4:**
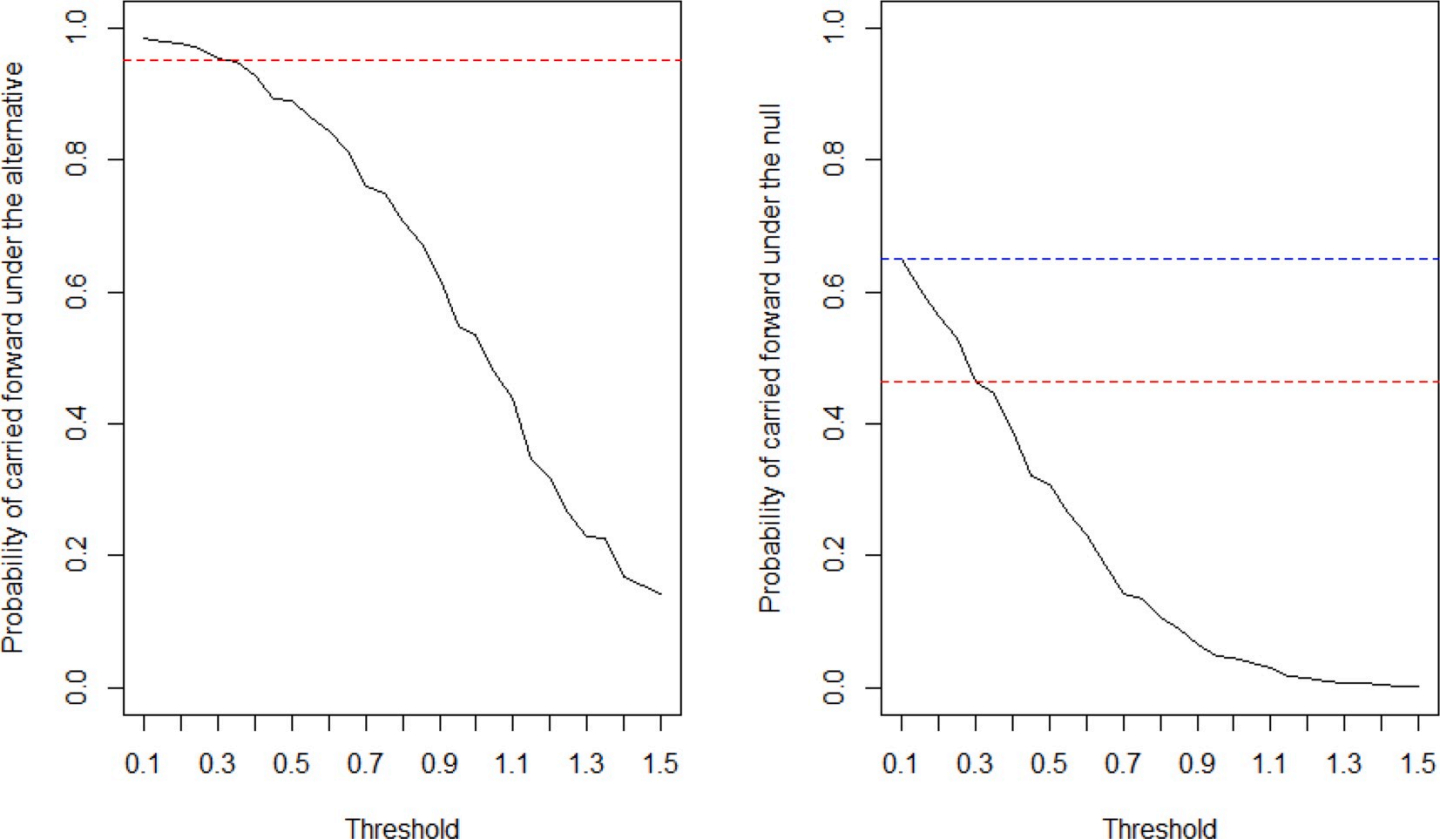
Probability of the study being carried forward to stage 2 under the alternative and under the null.

We evaluate one scenario of most interest from the study team: scenario 1, to demonstrate how a threshold can be selected for IA. When there exists underlying treatment effect, it should be detected and move forward into the second stage for completion at least 95% of the time. This choice of probability, i.e., 95% in this example, should be set reasonably high and at least higher than the desired overall statistical power of the study to provide adequate chance of success at the end of the study. Since the probability of moving the study forward is decreasing as the threshold becomes more stringent, the candidate for the threshold is narrowed down to the region of [0.1, 0.3] (see Fig. 4). On the other hand, if there is no treatment effect, the probability of carrying forward the trial should not be unreasonably high. As in Fig. 4, when using the threshold of 0.1, this probability is as high as 65%. With the threshold of 0.3, the probability drops to 46%. Therefore, we select 0.3 as the threshold for treatment group selection at IA.

A confirmatory study is often powered at 90%. Based on simulation, when the stage-1 sample size is fixed at 20 subjects per arm and there are 124 subjects per arm in the second stage that are preplanned, the overall power achieves 90%. The resulting total preplanned sample size is 368. Since the trial will be occasionally stopped for futility (5.1% of the time) under the considered effect size scenario, the expected sample size is 357 (under these assumed effect rates). Table 1 presents the operating characteristics of the simulated design for the study. The probability of selecting the high dose is substantially higher than the other active arms. Given entering stage 2, the probability of claiming significance at final analysis is as high as 93.7%.

**Table 1:** Conditional power, probabilities of arm selection and false stopping probability.

Conditional Power Given Carried Forward	Arm Selection			False Stopping Probability
93.7%	High Dose	Mid Dose	Low Dose	3.6%
	58.7%	31.7%	4.5%	

Another quantity that is worth checking is the false stopping probability. In the simulation, it reflects how often a trial is stopped for futility at IA but will achieve statistical significance at the final analysis (flip flop), had we moved the trial forward to the second stage. We want to see that this risk is small.

**Fig. 5:**
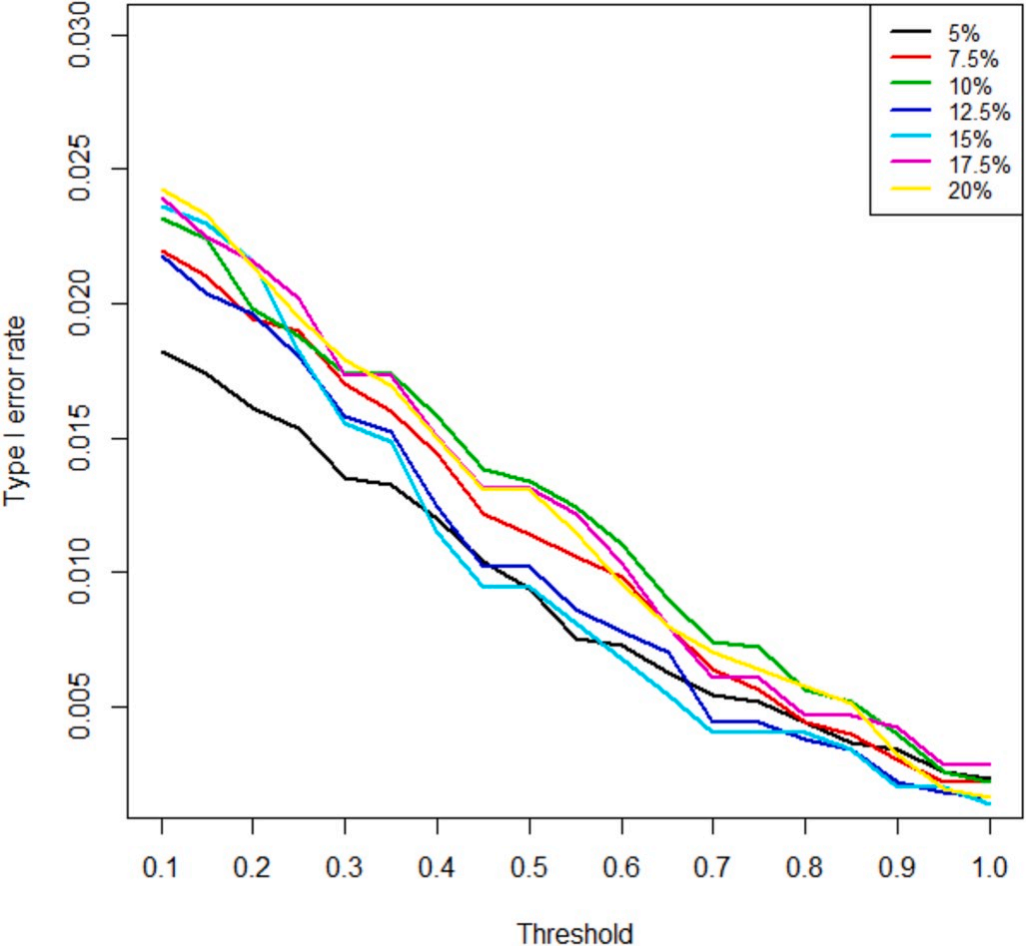
Type 1 error for various assumed event rates.

In addition, we also examined the type I error control through the simulation. We considered a global null hypothesis that each of the three dose groups has the same event rate as the placebo group for the primary endpoint. The reported rejection rates are defined as rejecting at least one null hypothesis. A range of event rates in the global null space are examined, namely, 5%, 7.5%, 10%, 12.5%, 15%, 17.5%, 20%. In Fig. 5, the type I error rates with various prespecified threshold values are presented for these assumed event rates and they are well controlled in the context of a test with overall level of 2.5%. It is recommended to examine more than one scenario based on the clinical background and interest. The combination test with closed testing procedure is known to control the FWER in the strong sense. Even though only one global null is considered in our simulations, one can extend to examine any configuration of null hypotheses based on clinical interest.

### 3.5 Comparing with a traditional design

Under the same treatment effect assumption, i.e., scenario 1, in a standard traditional non-adaptive design with four arms (three active arms and one control arm) and no IA, the sample size required is 91 subjects per arm (364 total) to achieve 90% power. To be consistent with the adaptive design that was illustrated earlier, the traditional design is using Dunnett's test and the power is defined as claiming significance for at least one active arm.

**Fig. 6:**
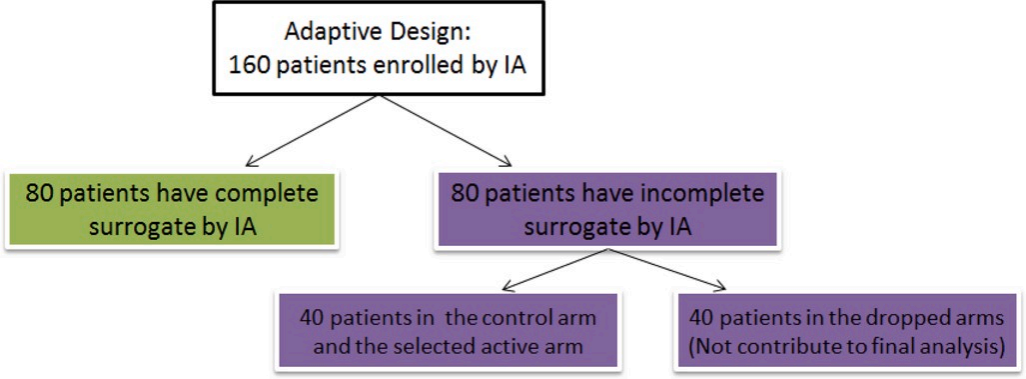
Schematic for the enrolled subjects by IA.

Compared with the average sample size of 357 in the proposed adaptive design, there seems to be very minimal saving in the sample size, slightly favoring the adaptive design. This is a common problem for every arm-dropping type of adaptive design with a delayed response. In our example, a further decomposition of the subjects enrolled by the time of IA reveals that 40 subjects (in the two arms that are dropped at IA) will end up not being included in the analysis (See Fig. 6). This is due to these subjects having incomplete surrogate endpoint observations at the time of IA.

There will be more savings in the sample size when surrogate endpoints mature faster and enrollment is slower. This suggests that adaptive designs such as this be considered only with relatively slower enrollment in relation to time to treatment maturation.

Nevertheless, the adaptive design is advantageous in that ethical considerations have a chance to be addressed. The stopping of groups due to any safety issues becomes possible in such adaptive designs, without undermining the validity of the statistical final analysis which protects type I error under the combination approach framework.

## 4 Discussion

In this article, we have presented an approach for simulating surrogate and primary endpoints at the design stage based on a Bayesian model which is built with the assistance of historical data. The Bayesian model is used to account for the variability in the relationship between an early surrogate endpoint and a late-occurring primary endpoint. This approach has the benefit of not requiring assumptions of concordance between the primary and surrogate endpoints, which is assumed in some trial design simulation software packages, and leads to better clarity of how the underlying correlation structure affects the ability of the surrogate to be used for IA decision purposes. The approach is illustrated through an example and compared with a traditional non-adaptive design. The savings in sample size and timeline depend on the length of period to observe the early surrogate endpoint, as well as the enrollment rate of the study. The proposed design with a model-based procedure in this article requires subject-level historical data to build the correlation model. However, there are circumstances where only the summary data are available. How to utilize study-level summary data, if provided, to explore the relationship between the surrogate and the primary endpoint can be a topic of future research. Summary data from multiple trials with similar subject population might be needed in such cases and must be properly handled with caution, especially for exchangeability assumptions and borrowing strength from each study.

The past several decades have seen an increase in the prevalence and usage of changing the endpoint of the analyses in clinical trials in both statistical and medical literature [32]. Even though such adaptive designs utilizing early surrogate endpoints may not have a clear advantage regarding statistical power, it allows an IA to facilitate the selection of a treatment early. Not only can inefficient treatment arms be dropped at interim evaluation, treatment due to safety concern also has a chance to be terminated. In addition, earlier decision-making on dose selection will benefit the investment and timeline of further development, e.g., for a second confirmatory study. Based on both efficacy and safety, as well as the prespecified adaptation rule, the Independent Data Monitoring Committee will provide recommendation about which treatment to move forward (or to terminate the study).

To gain insight on the relationship between the early surrogate endpoint and the primary endpoint in our example, a historical dataset of patients from a similar patient population is analyzed to ultimately guide the IA decisions in this approach. As more historical data become available, the approach can be extended to incorporate more information. However, our approach is limited to the setting where a historical dataset exists with assessments of both the primary endpoint and the surrogate. Further, the assumption about the correlation between these two endpoints must hold for both the control arm and the previously unobserved treatment arm. If the mechanism of action is different for the control (assuming it is an active control) and the experimental treatment, it may be possible that the correlation is different within each of the treatment arms. However, this assumption about the correlation is needed for any method for using a surrogate endpoint at an interim analysis to predict the outcome of the primary endpoint at the end of a study.

In describing our method, we used a simple linear model where the time-to-event primary endpoint was log-transformed. However, our method can be used with other models if the functional relationship between the two endpoints is not well captured with this linear model. Any appropriate model may be evaluated and used in the proposed Bayesian analysis. Please keep in mind that the clinical team may have difficulty understanding the correlation structure assumed between log(T) and X if they are only given the values of the parameters (a,b,σ). One way to aid in communication is to calculate the correlation directly when using a simple linear model. For nonlinear models, it should be explained that the correlation is more complicated.
